# Tracing key genes associated with the *Pinctada margaritifera* albino phenotype from juvenile to cultured pearl harvest stages using multiple whole transcriptome sequencing

**DOI:** 10.1186/s12864-020-07015-w

**Published:** 2020-09-25

**Authors:** Pauline Auffret, Jérémy Le Luyer, Manaarii Sham Koua, Virgile Quillien, Chin-Long Ky

**Affiliations:** 1 Ifremer, UMR EIO 241, Centre du Pacifique, BP 49, 98719 Taravao, Tahiti, Polynéise française France; 2 Ifremer, UMR LEMAR UBO CNRS Ifremer IRD 6539, ZI Pointe Diable CS 10070, F-29280 Plouzane, France; 3 IHPE, Université de Montpellier, CNRS, Ifremer, Université de Perpignan Via Domitia, F-34090 Montpellier, France

**Keywords:** *Pinctada margaritifera*, Albinism, Pigmentation, RNA-seq, Notch signaling pathway, Tyrosinase, Biomineralization

## Abstract

**Background:**

Albino mutations are commonly observed in the animal kingdom, including in bivalves. In the black-lipped pearl oyster *Pinctada margaritifera,* albino specimens are characterized by total or partial absence of colouration resulting in typical white shell phenotype expression. The relationship of shell colour with resulting cultured pearl colour is of great economic interest in *P. margaritifera,* on which a pearl industry is based. Hence, the albino phenotype provides a useful way to examine the molecular mechanisms underlying pigmentation.

**Results:**

Whole transcriptome RNA-sequencing analysis comparing albino and black wild-type phenotypes at three stages over the culture cycle of *P. margaritifera* revealed a total of 1606, 798 and 187 differentially expressed genes in whole juvenile, adult mantle and pearl sac tissue, respectively. These genes were found to be involved in five main molecular pathways, tightly linked to known pigmentation pathways: melanogenesis, calcium signalling pathway, Notch signalling pathway, pigment transport and biomineralization. Additionally, significant phenotype-associated SNPs were selected (*N* = 159), including two located in the *Pif* biomineralization gene, which codes for nacre formation. Interestingly, significantly different transcript splicing was detected between juvenile (*N* = 1366) and adult mantle tissue (*N* = 313) in, e.g., the tyrosinase *Tyr-1* gene, which showed more complex regulation in mantle, and the Notch1 encoding gene, which was upregulated in albino juveniles.

**Conclusion:**

This multiple RNA-seq approach provided new knowledge about genes associated with the *P. margaritifera* albino phenotype, highlighting: 1) new molecular pathways, such as the Notch signalling pathway in pigmentation, 2) associated SNP markers with biomineraliszation gene of interest like *Pif* for marker-assisted selection and prevention of inbreeding, and 3) alternative gene splicing for melanin biosynthesis implicating tyrosinase.

## Background

Mutations affecting pigmentation were among the first in the animal kingdom to be studied for Mendelian inheritance. Some species became ideal models for studying the genetic mechanisms that determine colour phenotypes [1]. Colour traits in domestic animals have been widely used as unique phenotypes for morphological broodstock selection and identification. In the aquaculture industry, body pigmentation can be an economically important trait selected to directly enhance the commercial value of a given species, for example, the red Nile tilapia (*Oreochromis niloticus*) compared with the wild type [2], or the majority of freshwater/marine ornamental species such as clownfishes [3]. Distinctive colouration characteristics, such as albinism, are common in most fish and shellfish species. For instance, in albino rainbow trout *Oncorhynchus mykiss*, the associated phenotype is characterised by a white or yellow skin according to the species [4]. Albino yellow catfish *Pelteobagrus fulvidraco* have been observed in control breeding in China [5]. Albino individuals have also been observed among aquacultured Russian sturgeons, one of the most valuable freshwater fish species worldwide [6]. In bivalves, white specimens of the Akoya pearl oyster *Pinctada fucata* are already used in selective breeding programs [7, 8]. Albinism can also be very useful as a visual phenotypic marker for experimental tagging studies [9]. In genetic studies, it allows the evaluation of chromosome manipulation efficiency in gynogenic or triploid animals [10] as well as the optimisation of gene editing approaches [11, 12].

Albinism is a genetically inherited trait that is usually defined as the absence or deficiency of pigmentation due to dysfunction in melanin production. Many mutations associated with albinism have been identified and well described in mice and humans (http://www.ifpcs.org/albinism/). Dysfunction of pigmentation has been found to result from multiple and complex molecular processes occurring during melanin production, transport and transfer and, for bivalves, in shell biomineralization. Many genes are involved along the pathway and have been reported to be deregulated in white-shelled bivalves [13–15]. Among these, tyrosinases are key enzymes regulating the melanin biosynthetic pathway. Molluscan tyrosinases are secreted from the mantle and transported to the prismatic shell layer where they contribute to melanin biosynthesis and shell pigmentation [16]. The Notch signalling pathway is an essential cell interaction mechanism, which plays a fundamental role in metazoan development [17]. Recently, the Notch signalling pathway was found to play a crucial role in shell pigmentation in the clam *Meretrix meretrix*, following a gene-dosage dependent pattern [15]. The calcium signalling pathway may also be implicated through activation of the Notch pathway in this species. Other studies implicated the Wnt signalling pathway in the maintenance of melanocyte and keratinocyte homeostasis and, therefore, its impact on pigmentation in the sea cucumber *Apostichopus japonicus* [18]*.* It is worth mentioning that the majority of the previous studies working on pigmentation in bivalves focused on mantle tissue samples.

Marine aquaculture of the black-lipped pearl oyster *Pinctada margaritifera* var. *Cumingii* for cultured pearl production is a major economic driver in French Polynesia for cultured pearl production (see [19] for current economic data). Cultured pearls form following a grafting operation, which consists in inserting a small piece of mantle tissue from a donor oyster (called a graft or *saibo*), into the gonad of a recipient oyster together with a spherical nucleus [20]. Nacre layers secreted by the mineralizing activity of the pearl sac (tissue which originated from the graft mantle) are progressively recovering the nucleus during culture time, forming the cultured pearl [21]. The whole process, from natural collection or hatchery production of spat, through juvenile growth to the adult stage at which the animals have reached a sufficient size to provide graft tissue or be grafted and finally the culture until pearl harvest, is a long process throughout which the biomineralization properties of specific tissues continue. *P. margaritifera* produces the largest range of cultured pearl colour in the genus [22, 23], including white pearls produced by the rare white albino shell morphotype, when these individuals are used as donors [24]. These albino individuals are characterised by total or partial absence of coloration resulting in a white shell (periostracum and calcitic layer) [14]. Studies on the *P. margaritifera* albino phenotype revealed Mendelian inheritance of shell colour under a three-allele model with no co-dominance, where the albino type is recessive to the black wild type and red phenotypes [24]. Suppressive subtractive hybridization (SSH) was previously used to characterise genes involved in shell colour by comparing black wild-type and albino phenotypes [14]*.* This earlier study revealed the implication of a small number of genes in albino phenotypes, including *ZINC,* a homolog of the *tyrosinase-related protein 1* gene, which codes for a protein with metallic zinc ion binding and a catalytic domain [14]*.*

In the present study, we investigated the multiple albino phenotype expression in *P. margaritifera* through successive and complementary whole transcriptomic sequencing analyses on different tissues from oysters of different stages collected over the entire pearl culture cycle. Three different tissues were analysed: whole flesh of juveniles, mantle tissue of donor oysters and, at harvest, the pearl sacs of recipients that had been grafted using albino saibo. Three distinctive RNA-seq approaches were then used to compare gene expression between the white shell phenotype (albino) and the black shell phenotype (wild type) in the same cohort. The three RNA-seq datasets shared only a few deregulated genes, underlining the importance of using multi-stage transcriptome analysis to provide a comprehensive overview of all processes potentially associated with the albino phenotype. We identified new candidate genes for white-shelled molluscs, together with genes already known to be expressed in the mantle tissue of *P. margaritifera*. Furthermore, we examined potential phenotype-associated SNPs and alternative gene splicing.

## Results

### Sequencing and mapping of libraries

The raw data of the three datasets were submitted to the National Center for Biotechnology (NCBI; BioProject ID: PRJNA642706). The sample identifiers were: 1) for the Juvenile (J) dataset: AN6 to AN10 (albino) and TB6 to TB10 (black wild-type); 2) for the Mantle (M) dataset: X11 to X15 (albino) and X6 to X9 (black wild-type); and 3) for the Pearl Sac (PS) dataset: B8, B11, B17 and B19 (albino) and N45, N47, N54 and N55 (black wild-type). A total of 41.14.10^9^ (J), 83.10^8^ (M), 5.61.10^8^ (PS) clean reads filtered from 1.17.10^9^ (J), 4.90.10^8^ (M), 5.41.10^8^ (PS) paired-end 100 bp raw reads were obtained from the selected samples. Mapping yield against the multi-tissue and stages reference transcriptome of *P. margaritifera* [25] reached 75.61% (J), 80.81% (M) and 74.17% (PS). Finally, a total 90.33% (J), 90.89% (M) and 91.27% (PS) of the mapped reads were kept after filtering (Table 1).

**Table 1:** General statistics of sequencing reads used in the three *P. margaritifera* RNA-seq datasets. Whole transcriptome sequencing libraries were generated on Illumina HiSeq4000 (paired-end (PE) sequencing 2 × 100 bp) and mapped to the reference transcriptome of *P. margaritifera.* The three datasets came from three different tissue compartments. Dataset ‘Juvenile’: Juvenile stage dataset (albino phenotype *N* = 5 and black wild-type phenotype *N* = 5). Dataset ‘Mantle’: Adult stage, mantle tissue (albino phenotype *N* = 4 and black wild-type phenotype *N* = 4). Dataset ‘Pearl Sac’: Adult stage, pearl sac (albino phenotype *N* = 4 and black wild-type phenotype *N* = 4)

Dataset	Juvenile	Mantle	Pearl Sac
PE raw reads (10^8^)	11.66	4.90	5.61
PE trimmed reads (10^8^)	11.37 (97.50%)	4.83 (98.58%)	5.41 (96.55%)
PE mapped reads (10^8^)	8.59 (75.61%)	3.90 (80.81%)	4.02 (74.17%)
PE filtered mapped reads (10^8^)	7.76 (90.33%)	3.55 (90.89%)	3.67 (91.27%)
Filtered counts (10^8^)	3.86	1.76	1.82
Filtered SNPs	388,500		–
Splicing events	1366	313	–

### Differential expression analysis

**Fig. 1:**
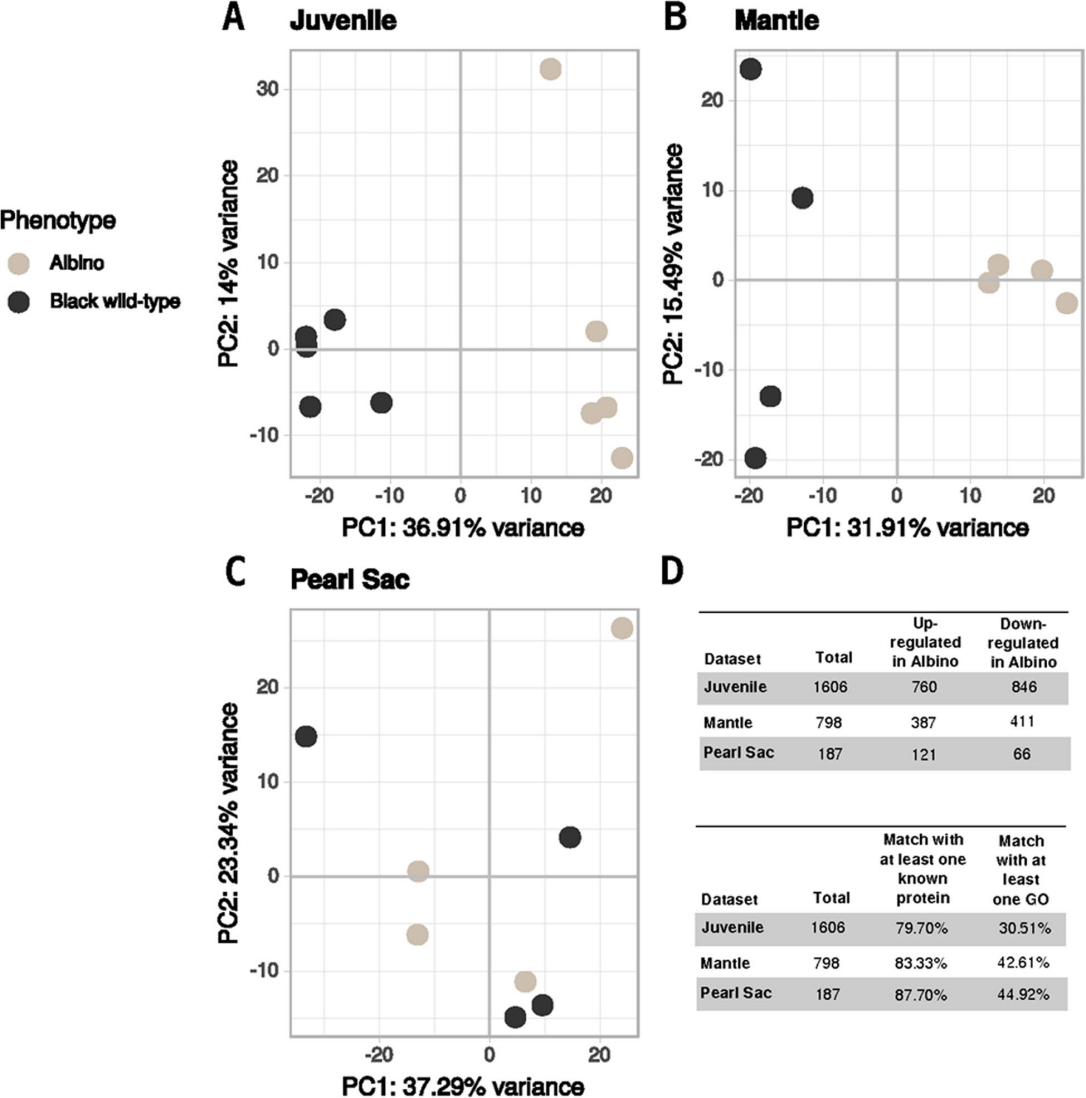
Principal component analysis (PCA) and differentially expressed genes (DEGs). PCA and DEGs between the *P. margaritifera* albino (white circle) versus black wild-type (black circle) phenotypes in the three tissue samples: Juvenile (**a**), Mantle (**b**) and Pearl Sac (**c**) datasets. The table (**d**) summarises the up- and down-regulated genes in the *P. margaritifera* albino phenotype

**Fig. 2:**
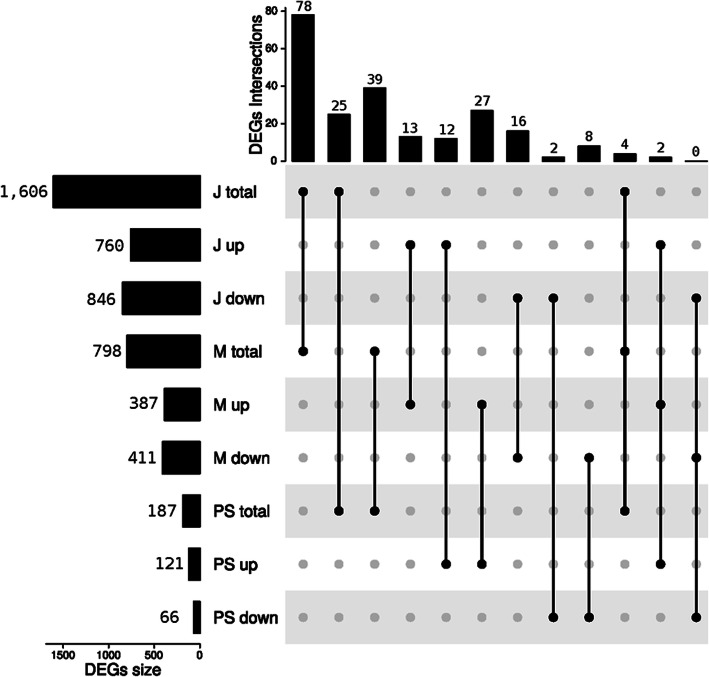
UpSet plot of differentially expressed genes (DEGs). The UpSet plot represents the intersections of DEGs between *P. margaritifera* albino versus black wild-type phenotype in three tissue compartments/datasets: juvenile (J), mantle (M) and pearl sac (PS). The number of total DEGs in the J, M and PS datasets are represented on the left (J total, M total, PS total) as are the number of up-regulated (J up, M up, PS up) and down-regulated genes (J down, M down, PS down) in the albino phenotype, as separate gene sets. The sizes of the intersections between these different sets are represented on the top barplot. The corresponding intersection is indicated by the connected dots

After filtering for residual expression, we retained 37,466; 28,286 and 32,313 transcripts in J, M and PS, respectively, for downstream analyses. By comparing albino with black wild-type results, a total of 1606 (J), 798 (M) and 187 (PS) differentially expressed genes (DEGs) were detected. Among these DEGs, 760 (J), 387 (M) and 121 (PS) were up-regulated in albino individuals, while 846 (J), 411 (M) and 66 (PS) were down-regulated (Additional file 1). There were notably fewer DEGs in the PS dataset. The principal component analysis (PCA) scatterplots show how the samples clustered according to their transcriptomic profiles (Fig. 1). J and M datasets PCAs were able to discriminate the albino phenotype from black wild-type samples along the first principal component, accounting for 36.91% (J) and 31.91% (M) of the total variance. In the PS dataset PCA, no clustering of samples according to phenotype can be observed (Fig. 1). Out of the total number of DEGs, 79.70% (J), 83.33% (M) and 87.70% (PS) had at least one match with a known protein and 30.51% (J), 42.61% (M) and 44.92% (PS) had at least one associated GO (Additional file 1). The number of DEGs shared among the three datasets are displayed in an UpSet plot in Fig. 2. Interestingly, a high proportion of common DEGs in pairwise comparisons of the three datasets were not regulated in a consistent way: 62.82% (M & J), 10.26% (M & PS), 44.00% (J & PS) and 50.00% (M & J & PS). Only four DEGs overlapped the three datasets, of which two were consistently up-regulated in the albino phenotype and only one had a predicted function (endonuclease enzyme) in the available annotation (Additional file 2).

### DEG enrichment analysis

We performed gene ontology (GO) enrichment analysis on the identified DEGs to look into the main molecular mechanisms possibly involved in albinism in the different tissues (Additional file 3). When summarised separately into semantic-based clustering treemaps with the reduce visualize gene ontology tool (REVIGO), enriched GO in the J, M and PS datasets showed few or no overlapping Molecular Functions (MF). GO analysis based on down-regulated DEGs revealed some relevant MFs in M dataset such as ion transmembrane transporter activity, pigment binding, calcium ion binding (Additional file 4: Fig. S1). When considering common down-regulated DEGs between M and J, the GO analysis highlighted the Notch binding function (Additional file 4: Fig. S1). KEEG automatic annotation server (KAAS) results showed relevant Kyoto encyclopedia of genes and genomes (KEGG) pathways. Two DEGs in the J dataset were involved in ABC transporters, as was one DEG in the M dataset. We also noted some key genes encoding proteins involved in several important signalling pathways (sp), such as calmodulin (*k02183*), cell division control protein 42 (*cdc42*, *k04383*) and RTK (epidermal growth factor receptor, *k04361*), among others. These are involved in RAS sp., Rap-1 sp., MAPK sp., calcium sp., melanogenesis (Additional file 4: Fig. S2). Notch sp. was also pinpointed in J and M (Additional file 4: Fig. S2).

### Identification of features of special interest

From the annotated DEGs (Additional file 1), five molecular pathways involved in pigmentation were selected:

#### Melanogenesis and *Tyr* genes encoding tyrosinase

Fifty-four tyrosinase-related transcripts were identified in *P. margaritifera* reference transcriptome, of which 31 were homologous to Tyrosinase-1 (*Tyr-1*), 13 were homologous to Tyrosinase-2 (*Tyr-2*) and 10 to Tyrosinase-3 (*Tyr-3*) (Additional file 5). No Tyrosinase-related genes were present in the PS DEGs dataset. In J samples, up-regulation of DEGs in the albino phenotype was observed for one *Tyr-1* (log2FC = 1.62) and one *Tyr-2* (log2FC = 1.76) homolog. In M tissue, up-regulation of DEGs in the albino phenotype was observed for one *Tyr-1* (log2FC = 3.32) and one *Tyr-3* (log2FC = 2.00) homolog, whereas down-regulated DEGs included four *Tyr-1* (log2FC ranging from − 3.48 to − 2.26) homologs, none of which corresponded to those DEGs found in the M dataset (Additional file 1). Moreover, in the M dataset, one P protein-related gene was down-regulated in the albino phenotype (log2FC = − 2.13).

#### Calcium signalling pathway

Two calmodulin-like proteins were up-regulated in albino phenotype in the M dataset (log2FC = 3.22 and 2.49). One was found down-regulated in the J dataset (log2FC = − 1.95).

#### Notch signalling pathway

One Notch-related gene was found up-regulated in the albino phenotype in the J dataset (log2FC = 3.06) and one Frizzled-related gene was down-regulated in the M dataset (log2FC = − 4.59).

#### ABC transporter family

One abcb gene was found up-regulated in the albino phenotype in the M dataset (log2FC = − 1.66) and one down-regulated in the J dataset (log2FC = 2.15). Two abcc genes were found down-regulated in the M dataset (log2FC = − 1.55 and log2FC = − 3.01) and one up-regulated in the J dataset (log2FC = − 2.95).

#### Biomineralization genes

One Pif gene was down-regulated in the M tissue (log2FC = − 2.28) and one shell protein was up-regulated in PS (log2FC = 2.70 and 2.54). Two serine protease inhibitor genes were deregulated in both M (log2FC = − 2.03 and 1.85) and PS (log2FC = 3.85). Three perlucin (log2FC from − 2.92 to 3.62) and four perlucin-like protein encoding genes (log2FC from − 1253 to 2.97) were deregulated in J; one perlucin (log2FC = 3.25) and four perlucin-like protein encoding genes (log2FC from − 4.16 to − 3.06) in M; one perlucin-like protein (log2FC = 2.72) in PS; one asparagin-rich protein (prism protein) in PS samples (log2FC = 2.70); and one zinc metalloproteinase encoding gene in J tissue (log2FC = 2.86) and two (log2FC = 5.97 and − 2.83) in M samples.

### SNP outlier analysis

Population genetics analysis showed a strong family effect in the J and M datasets with maximal group assignment probability (Additional file 4: Fig. S3). When merged, variant calling files of J and M contained 388,500 SNPs located on 19,772 different transcripts. Of these, the lfmm analysis returned 261 (0.07%) significant phenotype-associated SNPs located on 170 different transcripts. PCAdapt detected 50,083 (12.89%) outliers. There were 159 candidate SNPs, i.e., common SNPs among outliers detected with PCAdapt and phenotype-associated SNPs detected with lfmm (Additional file 6). These were located on 115 different transcripts and 92 of them were annotated. Among these, two were non-synonymous substitutions affecting predicted proteins ‘periostin’ (Q29XZ0) and ‘short-chain collagen C4’ (P18503). Interestingly, two candidate SNPs were located on the *Pif* gene and were synonymous. There were two common transcripts between the concatenated M and J DEG set and the common outlier SNP set. Only one common transcript was annotated (probable RNA-directed DNA polymerase from transposon BS reverse transcriptase) in the reference transcriptome.

### Alternative gene splicing and exon usage

**Fig. 3:**
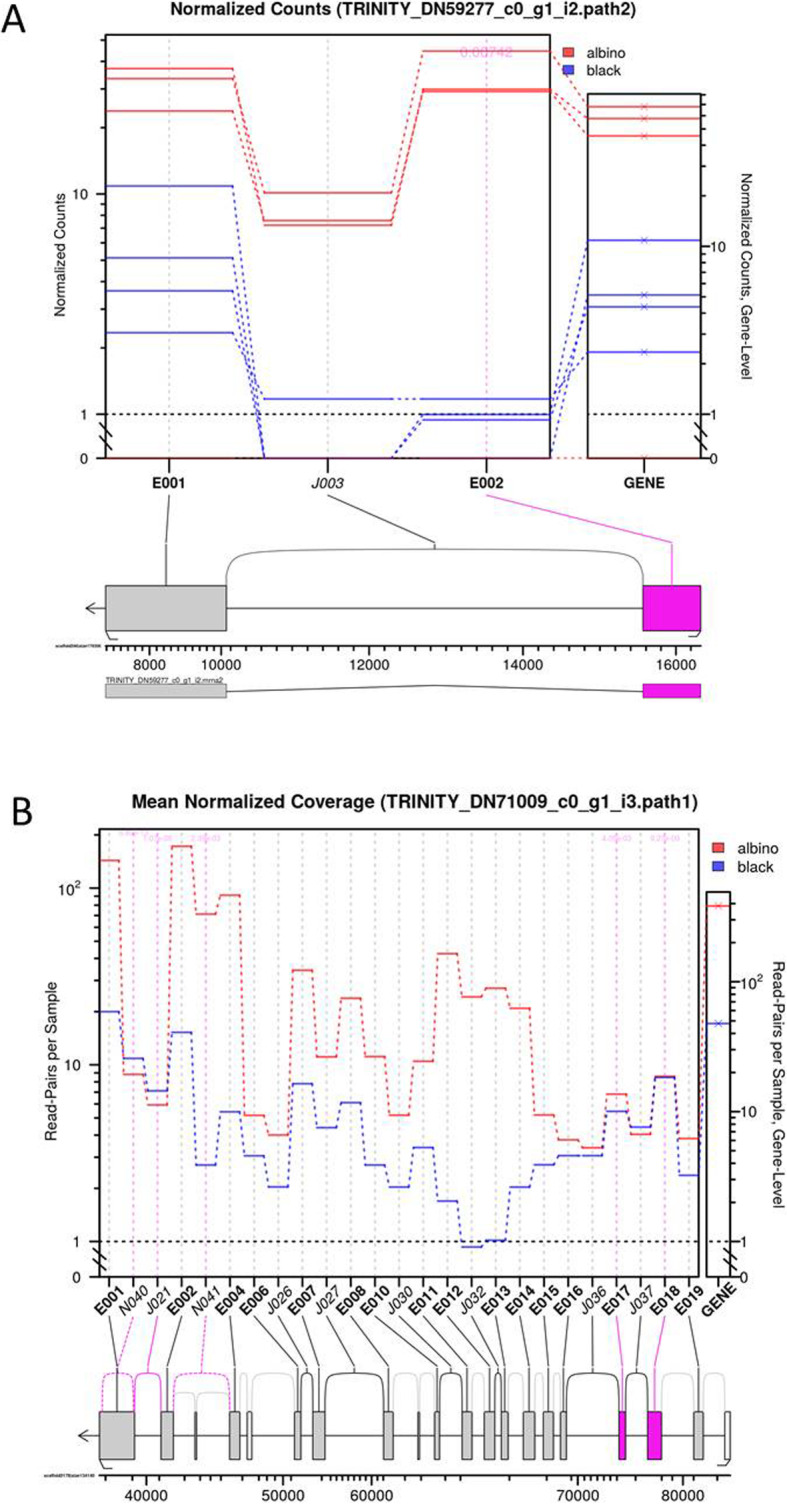
Splicing event for two genes of *P. margaritifera*. Tyrosinase-1 in the mantle (**a**) and Notch homolog protein 1 in juvenile (**b**). Normalised counts are plotted for each gene section, either exon (E) or junction (J), and each individual (black wild-type in blue and albino in red). Values in the box plot represent *p*-values (Fisher’s test) for each gene section

Significant differential splicing was observed for 1366 (J) and 313 (M) transcripts (adjusted *p* value < 0.01, Additional file 7). Of these, 1043 (J) and 251 (M) involved annotated transcripts. Some potentially interesting genes known to be involved in biomineralization and/or pigmentation processes were identified, such as prism shell proteins, ABC transporter family members, calmodulin, collagen-alpha chain members, neurogenic locus notch homolog protein 1, PDZ, perlucin, Pif, putative Tyr-1, and Ras-related proteins in J; and ABCb transporter, one mantle protein encoding gene, Pif, Tyr-1 and zinc metalloproteinase in M. Differential splicing events were found to be deregulated for 21 (J) and 16 (M) transcripts, including Tyr-1 (Fig. 3) and Zinc metalloproteinase in M, and Notch in J (Fig. 3). There were 73 common transcripts between the J and M datasets showing significant differential splicing, among these, the Pif transcript carried two synonymous candidate SNPs.

## Discussion

Over the past decade, the advent of next-generation sequencing technologies has allowed significant progress to be made in understanding the genetic bases and molecular mechanisms involved in phenotypic trait expression in non-model species such as *P. margaritifera*. Previous studies used molecular markers or whole transcriptome to investigate pearl quality, growth, dynamics of nacre layer formation and biomineralization [25–27]. In this study, we used RNA-seq to explore the molecular bases of the albino phenotype and to identify genes and molecular pathways associated with colour mutation across culture stages, from juvenile to adult, and tissue types (whole juvenile, mantle graft and pearl sac). We used independent multi-parental experiments to explore the genetic basis (SNPs), gene expression and alternative splicing underlying albinism in *P. margaritifera*. Our results show strong tissue and/or stage-specific responses, as suggested by the relatively low number of common DEGs across datasets. However, the related biological functions associated with the albino phenotype were redundant across J and M datasets and provide unprecedented information on the molecular effectors of albino pigmentation in *P. margaritifera*. Indeed, previous work investigating the genetic basis of rare flesh and shell colour variant polymorphism in *P. margaritifera* showed that shell colour is controlled by a three-allele model where albino is recessive to black wild type and not related to environmental effects [24]. Here, the use of three datasets from different tissues and representing different developmental stages adds value to the study, as the shell pigmentation of *P. margaritifera* is perceptible from the juvenile stage (three months old) [22] and can be related to pearl colour.

### The notch signalling pathway, involved in shell formation and pigmentation, was impacted in juveniles

The Notch signalling pathway is a highly conserved cell signalling system that regulates processes such as cell growth and proliferation, cell fate decisions, differentiation and stem cell maintenance [17]. The roles of the Notch signalling system have been well described in development [17] and mammalian hair pigmentation, where it is involved in the maintenance of melanoblasts [28]. Recently, it has been shown that the Notch signalling system is activated in a pearl oyster allograft (donor and recipient oysters from the same species) where it helps suppress the induced inflammation response [29]. In the clam *M. meretrix*, it was suggested that the Notch signalling pathway plays an important role in shell pigmentation following a gene dosage-dependent pattern and was also potentially involved in the shell colour patterning [15]. The Notch signalling pathway has also been reported to play a role in the pigmentation process in Pacific oyster *C. gigas,* where it was proposed that white-shelled oysters used endocytosis to down-regulate Notch level, thus causing melanoblast apoptosis, which prevents pigmentation [13]. Finally, in the sea cucumber *Apostichopus japonicus*, the Notch signalling pathway might be an upstream component of the pigmentation process by determining the location and boundaries of pigment occurrence [18]. Here we showed that the neurogenic locus Notch homolog protein 1 (Notch1), is up-regulated in albino Juvenile phenotype *P. margaritifera* individuals. These results strongly support the early role of Notch signalling regulation, which most likely results in the inhibition of shell pigmentation. Interestingly, in *Crassostrea gigas,* authors found Notch2, but not Notch1, to be down-regulated in white-shelled oysters [13]. Notch1 and Notch2 are the two closest related Notch paralogs with opposite biological functions to each other in mammals [30]. We further show differential exon usage of the same Notch1 gene, but no related SNPs in coding regions. Putative splice variants of Notch were also identified in the butterfly *Vanessa cardui* and suggested to be part of a conserved Notch signalling cassette. Certain of these genes were down-regulated during pupal stages, and their activation during the pre-ommochrome stage might play a role in pattern positional information on the wing [31]*.* Admittedly, further studies will be needed to assess the precise role of alternative splicing in the Notch signalling pathway and any subsequent impact on shell pigmentation in *P. margaritifera.* In bivalves, shell construction starts early in larval development and differences in pigmentation can already be seen at the juvenile stage (three months old) [22]. Previous studies on shell pigmentation in *P. margaritifera* have, however, only focused on adult specimens. Here, the inclusion of juvenile specimens in our design allowed us to see the specific impact of the Notch signalling pathway in earlier shell formation and pigmentation.

### Adult mantle and juvenile transcriptome analysis highlights the association of key genes involved in melanogenesis with the *P. margaritifera* albino phenotype

Tyrosinase is a key copper-containing enzyme known to be involved in shell structuration and pigmentation in bivalve molluscs through a large spectrum of biological processes, including pigment production, innate immunity, wound healing and exoskeleton building and hardening [32]. Tyrosinases are, for example, essential in the melanogenesis pathway as key regulators of melanin biosynthesis [33]. Tyrosinase deregulation has been reported in the bivalves *C. gigas*, *P. yessoensis* and *P. fucata martensii,* which show contrasting pigmentation phenotypes [13, 34, 35]. In bivalve molluscs, mantle tissue secretes proteins responsible for shell calcification and pigmentation, and pigments are probably formed in the secretory cells in the mantle and incorporated into the shell along the growing edge [35]. It is thus not surprising that expression of Tyrosinase genes in *P. margaritifera* occurs predominantly in the mantle tissue [32]. In this study, no *Tyr-2* was found among the mantle DEGs, which is consistent with a previous study on *P. margaritifera* [14]. *Tyr-1*, however, showed isoform-specific regulation in mantle (one up-regulated and four down-regulated). We identified 54 tyrosinase or tyrosinase-like protein encoding genes in *P. margaritifera* reference transcriptome [25]. A complex evolutionary history has been described for tyrosinase genes in bivalves, in which tyrosinase duplicates may have been retained because of their functional diversification in the mantle [32]. Further studies will, therefore, be needed to examine more closely any isoform-specific function/s of the *Tyr* gene family in *Pinctada* sp. We also observed down-regulation of the P protein-encoding gene, a key gene involved in melanogenesis, in the albino phenotype mantle of *P. margaritifera*. P protein, known as the pink-eyed dilution protein encoded by the *OCA2* gene, could be involved in the transport of tyrosine, the precursor to melanin synthesis, within human melanocytes. Mutation in this protein may cause complete or partial albinism [36]. To our knowledge, this is the first report of such a gene in bivalves. Moreover, *frizzled-3* was also found to be down-regulated in the mantle of the *P. margaritifera* albino phenotype. Frizzled is an essential receptor associated with the Wnt signalling pathway, which is involved in the maintenance of melanocyte and keratinocyte homeostasis [18]. Our result is consistent with a previous study in zebrafish where inhibition of the Wnt signalling pathway decreased pigment cells [37]. We also observed deregulation of members of the ATP-binding cassette (ABC) transporter family, which is a large transporter family related to cellular detoxification, observed in all kingdoms of life [13, 38]. It has notably been detected in numerous marine species, including bivalves. In oysters, the involvement of *Abcb1* has been hypothesised in a molecular detoxification system through the transport of a variety of molecules and xenobiotics across the cell membrane [39]. Interestingly, in *C. gigas*, ATP-binding cassette members might be responsible for the transportation of pigment-related substrate that influences pigmentation [13]. Moreover, in human, calcium signalling plays a role in the co-regulation of retinal pigment epithelial cells [40]. In the present study, we showed that the calcium signalling pathway, especially *Calmodulin* gene expression, is associated with the albino phenotype in adult and juvenile *P. margaritifera*. Calmodulin and calmodulin-like proteins are involved in biomineralization and melanogenesis in bivalve molluscs [27]. Our results thus suggest that the albino white shell phenotype in *P. margaritifera* may result from dysfunction of melanin transfer from the mantle to the shell.

### Multiple transcriptome analysis confirms that shell pigmentation requires essential biomineralization genes

A previous study investigated the role of a subset of genes in shell biomineralization and pigmentation of *P. margaritifera* by comparing black wild-type and albino individuals [14]. Our present results are concordant for key biomineralization genes, including *Pif* in mantle and shell matrix proteins in pearl sac, but others are deregulated inconsistently, like serine protease inhibitor in mantle and pearl sac. We also report the deregulation of genes for perlucin and perlucin-like protein in pearl sac, both of which are involved in shell biomineralization [41]. The Pif gene codes for an important acidic matrix protein known to be involved in nacre formation and has a complex role in biomineralization [42]. Here, *Pif* shows significant exon usage in both J and M datasets, and also carries two significant synonymous phenotype-associated SNPs. These results suggest a tight association of *Pif* with the albino phenotype in M and J, but the elucidation of its precise role in *P. margaritifera* biomineralization, which has already been shown to be complex [25], would require further investigation. Finally, we observe limited albino specific signature of in PS tissue genes expression. These results possibly suggest that PS response is significantly affected by the PS environment, *i.e* recipient oyster activity, supporting previous elegant work based on xenograft experiments and final pearl quality [43].

An understanding of the molecular and genetic basis of the *P. margaritifera* albino phenotype would be a valuable resource for its aquaculture in French Polynesia. Indeed, Polynesian pearl production is currently and mainly focused on dark-coloured pearls. Developing the production of lighter-coloured pearls could open new market opportunities for the Tahitian pearl industry. In fact, albino donor grafts into wild-type recipients produce light silver, white or light cream cultured pearls, with some interesting pinkish and blue reflected lustre. As albino pearl oysters are very rare in French Polynesia*,* setting up selection programs will require reliable genetic markers (not only limited to coding-regions) to monitor the level of inbreeding throughout production.

## Conclusions

This study was designed to identify key genes associated with the *P. margaritifera* albino phenotype, from juvenile to cultured pearl harvest stages, using comparative transcriptome analysis with black-shelled wild-type individuals. Results of the transcriptome analysis in three different datasets: juvenile whole flesh (J), adult mantle (M) and pearl sac (PS) revealed quite specific transcriptomic signatures with some related functions. We successfully identified a set of genes involved in pigmentation, including several genes not previously described in pearl oyster *P. margaritifera*. The study also showed the putative association of significantly different exon usage and SNPs in the albino phenotype. For the first time in *P. margaritifera*, the involvement of the Notch signalling pathway in pigmentation was highlighted. *Notch1* was specifically deregulated and showed alternative splicing in the J dataset, suggesting an early role of this gene in shell formation and pigmentation. We confirmed the association of genes involved in melanin biosynthesis and transport, such as calmodulin and tyrosinase, with putative associated alternative splicing for the latter. We also discussed the possible involvement of P protein, hitherto only described in mammal albino phenotypes. This analysis also corroborates previous studies by calling into question the association of biomineralization genes like *Pif*, which carried two synonymous phenotype-associated SNPs. Future studies should further investigate the role of alternative splicing and SNPs using a complete annotated genome (presently under construction). The potential involvement of non-coding RNA in shell pigmentation should also be explored in *P. margaritifera.* Similarly, complementary approaches based on metabolites secretion would provide further evidence and validate regulatory network related to pigment synthesis in oyster.

## Methods

### Animal tissue sampling

Three datasets were obtained from different tissue compartments, comparing two families with white albino and black wild-type phenotypes at three key stages of pearl production/ culture. These two families were both F1, produced simultaneously in a hatchery system at Ifremer facilities, following routine rearing procedures described previously [44, 45]. Juveniles and adults were reared according following standard procedures for *P. margaritifera* farming [46]. The F1 families were produced from multi-parental crosses made with wild broodstock originating from Takume atoll (Tuamotu archipelago, French Polynesia). After sampling, all tissues were preserved in ribonucleic acid (RNA) later (Quiagen) and kept at − 80 °C until RNA extraction. The three samplings from the juvenile stage until pearl harvest were made over a four-year period encompassing the whole pearl culture cycle.

**Fig. 4:**
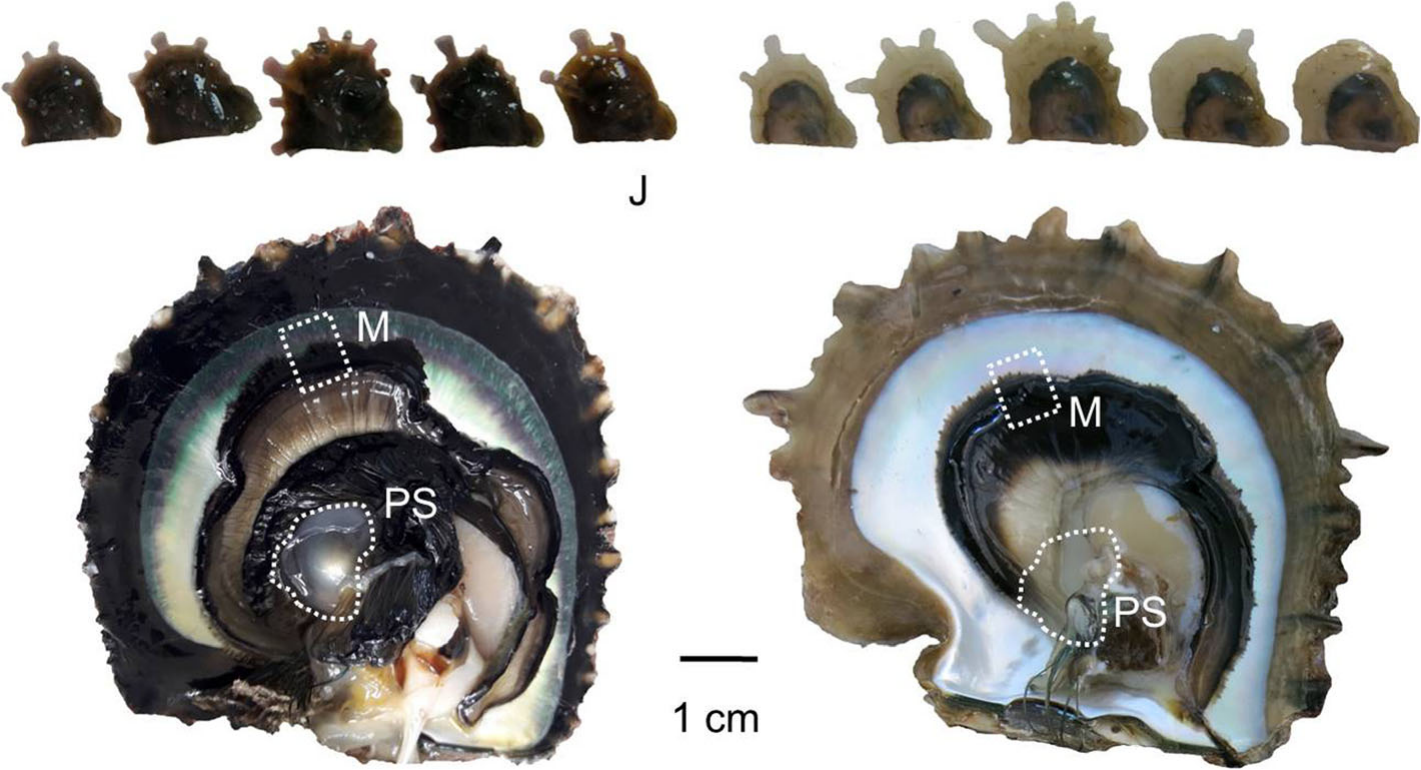
*P. margaritifera* samples: 1) Juvenile stage (J dataset) with albino phenotype (*N* = 5) and black wild-type phenotype (*N* = 5), 2) Mantle tissue (M dataset) and 3) pearl sac (PS dataset). For M and PS, the picture shows a black-shelled wild-type example. All pictures were made by the corresponding author C.-L. Ky, with its permission for publication in this journal

Dataset 1 Juvenile stage (J) (Fig. 4). *P. margaritifera* juveniles of 4.5 months were sampled (albino phenotype *N* = 5; black wild-type phenotype *N* = 5). Tissue samples consisted of whole soft tissues, i.e., entire animals

.

Dataset 2 Adult stage, mantle tissue (M) (Fig. 4). Tissue samples consisted of fragments of mantle (albino phenotype *N* = 4; black wild-type phenotype *N* = 4).

Dataset 3 Adult stage, pearl sac (PS) (Fig. 4). Eighty months before this sampling, recipient oysters had been grafted using a set of white albino and black wild-type donors using a classical experimental graft designed as previously described [47]. At pearl harvest, pearl sacs were sampled to provide tissue for this dataset (albino phenotype *N* = 4 and black wild-type phenotype *N* = 4).

### RNA extraction and sequencing

For all samples, total RNA was extracted with TRIzol Reagent (Life Technologies) at a ratio of 1 ml per 100 mg tissue following manufacturer’s recommendations. RNA quantity/integrity and purity were validated on a Nanodrop (NanoDrop Technologies Inc.) and a BioAnalyzer 2100 (Agilent Technologies), respectively. RNA was dried in RNA-stable solution (ThermoFisher Scientific) following manufacturer’s recommendations. Samples were shipped at room temperature to McGill sequencing platform services (Montreal, Canada). TruSeq Sample Prep. (Illumina, San Diego, California CA, USA) RNA libraries were sequenced on a HiSeq 4000 100-bp paired-end (PE) sequencing device.

### Differential expression analysis

Read quality was assessed with fastqc v0.11.5 [47] and multiQC [48]. Raw reads were filtered to remove sequencing adapters and for quality trimming (Q = 28) using: (*i*) Cutadapt v1.13 [49] for the M dataset and (*ii*) Trimmomatic v0.36 [50] for the PS and J datasets. For each dataset, only surviving paired-end reads were retained. Filtered reads were mapped on the multi-tissue reference transcriptome of *Pinctada margaritifera* (41,075 contigs) [25] using bwa v0.7.15 [51] with standard parameters. Reads with low mapping quality (Q ≥ 5), mispairing or multi-mapping were removed using Samtools v1.4.1 [52]. A matrix of raw counts was built using HTSeq-count v0.6.1 [53]. To minimise the false-positive rate, count matrices were filtered for little expressed transcripts. For each dataset, all transcripts with less than 10 counts in at least two samples were discarded. We identified differentially expressed genes (DEGs) between albino and black wild-type individuals using DESeq2 v1.16.2 [54] with R v3.4.0 (https://www.R-project.org/) following the standard workflow. The DESeq2 method internally corrects for library size and uses negative binomial generalised linear models to test for differential expression. In this study, the statistical models were built using ‘counts ~ Phenotype’ design formula, where the Phenotype qualitative variable indicates oyster phenotype (albino/black wild-type). All features with absolute log2 fold change greater than 1.5 and adjusted *p*-value smaller than 0.05 (Benjamini-Hochberg method) were reported as differentially expressed (DEGs). Overlapping DEGs between the three datasets were visualised using the UpSet function from ComplexHeatmap R package version 2.0.0 [55]. For this study, prior genes annotation with blastx (e-value 10–5 [25];) against Swissprot-Uniprot, has been supplemented with searches against conserved protein domain databases. First, longest Open Reading Frame (ORF; min. Length 100 amino acid) for each gene was computed with TransDecoder v5.5.0 (https://github.com/TransDecoder/TransDecoder) tools. ORF where scan for protein conserved domains against CDD, TIGRFAM, PRINTS, SMART, SUPERFAMILY and Hamap databases using InterProScan v5.28–67.0 [56] and GO were retrieved using interPro2GO database (version 2020/04/18; http://www.geneontology.org/external2go/interpro2go). This search resulted in a total of 16,463 matches; out of which 9360 had an associated GO entry and 932 had no prior GO associated using Swissprot only. To test the overrepresentation of gene ontology (GO) terms in resulting DEGs, we used Goatools v0.8.4 [57] through the ‘go_enrichment’ pipeline (https://github.com/enormandeau/go_enrichment) with the go-basic.obo database (release 2018-05-07). The resulting lists of significant GO enriched terms were filtered for Fisher’s Test *p*-value < 0.05 and used for semantic-based clustering using REVIGO with an allowed similarity of 0.5 [58]. DEG lists were submitted to the KAAS server ([59], last updated: April 3, 2015) to visualise related KEGG pathways.

### Population genetics analysis

In the J and M datasets, we investigated sites that were variable between the white albino and black wild-type populations. We did not include the PS dataset in this part of the study because of possible contamination with recipient RNA during pearl sac sampling. Single nucleotide polymorphisms (SNPs) were called from pre-processed aligned reads using Freebayes v1.1.0–3-g961e5f3 [60] with a required minimum mapping quality of 20. Pre-processing of aligned reads included marking and removing duplicates, correcting N cigar reads, sorting and indexing bam files using gatk v4.0.2.1 [61] and the Picard tools suite v1.119 [62]. Resulting variant calling files (VCF) were filtered for missing data and indels (none authorised), allele frequency (≥ 0.1) and depth (≥ 20) using vcftools v0.1.14 [63]. Filtered VCF for J and M were merged to minimise family effects and focus on phenotype-associated events. To investigate the structure of albino and black wild-type individuals within the two populations, we performed a population genetics analysis on the filtered VCF files using the following R packages: vcfR v1.8.0 [64], adegenet v2.1.1 [65] and genepop v1.0.5 [66] with R v3.4.0. We then looked for outlier SNPs (adjusted *p* value < 0.01, Benjamini-Hochberg procedure) with PCAdapt v4.0.3 [67] PCAdapt tests for outliers using correlations between SNPs and first principal components (K) of PCA analysis. We used K = 2 (J and M separately) and K = 3 (M and J merged) and adjusted *p*-value < 0.01, according to the Benjamini-Hochberg procedure. We also performed phenotype-associated SNP analysis using lfmm v0.0 (https://bcm-uga.github.io/lfmm/index.html) to find potential genetic markers of albinism. The Lfmm program constructs latent factor mixed models (LFMMs), which are statistical regression models to test associations between a multidimensional set of response variables (here, genotypes) and a variable of interest (here, phenotype). LFMMs include unobserved variables, called latent factors, which correct the model for confounding effects due to population structure and other hidden causes. We selected phenotype-associated SNPs according to adjusted *p* values < 0.01. Phenotype-associated SNPs were annotated using the ‘*LongOrfs*’ function implemented in Transdecoder v3.0.1 (https://github.com/TransDecoder/TransDecoder/wiki) and personal python script.

### Alternative gene splicing and exon usage

To detect differential splicing events in the three RNA-seq datasets, the filtered reads were mapped on the draft genome of *P. margaritifera* using GSNAP aligner v2017-03-17 [68], allowing five mismatches, splicing and using the ‘*splitting-output*’ function to retain only concordant and unique mapped paired-end reads, as described previously [25]. We used the QORTs [69] and JunctionSeq R packages [70] to detect significant differences in exon usage. Only exons and junctions with a minimal coverage of six were used for the analysis and only differences with FDR < 0.01 were considered significant.

## Supplementary information

**Additional file 1. **Lists of DEGs between the *P. margaritifera* albino phenotype and black wild-type phenotype, associated statistics and annotation for the three datasets (Juvenile, Mantle and Pearl Sac).

**Additional file 2. **Lists of common DEGs between the *P. margaritifera* albino phenotype and black wild-type phenotype overlapping the three datasets (Juvenile, Mantle and Pearl Sac).

**Additional file 3. **Lists of enriched GO terms in the *P. margaritifera* albino phenotype compared with the black wild-type phenotype in the three datasets (Juvenile, Mantle and Pearl Sac).

**Additional file 4: **(.docx) **Fig. S1.** Summarised REVIGO treemaps plot for gene ontology enrichment analysis between the *P. margaritifera* albino versus black wild-type phenotypes. **Fig. S2.** Signalling pathways (sp) potentially impacted by genes deregulated in albino *P. margaritifera* compared with the black wild-type. **Fig. S3.** Composition plots of albino and black wild-type populations of pearl oyster *P. margaritifera* based on filtered SNPs called with Freebayes in both Juvenile and Mantle datasets.

**Additional file 5. **List of tyrosinase-related transcripts in *P. margaritifera* reference transcriptome.

**Additional file 6.** List of 159 candidate SNPs, i.e., common SNPs among outliers detected with PCAdapt and phenotype-associated SNPs detected with lfmm, based on filtered SNPs called with Freebayes in both Juvenile and Mantle datasets.

**Additional file 7. **List of transcripts showing significant differential splicing (adjusted *p* value < 0.01) in Juvenile and Mantle datasets.

## Data Availability

The data for the genome of *P. margaritifera* is available following the direct web link: https://sextant.ifremer.fr/eng/Donnees/Catalogue#/metadata/0d2dda11-db53-43f5-b0d4-1acb2ca0bdc4. The full name of the data is: “Draft genome assembly of *Pinctada margaritifera*”*.* For the multi-tissue reference transcriptome of *Pinctada margaritifera*, theTranscriptome Shotgun Assembly project has been deposited at DDBJ/EMBL/GenBankunder the accession GIUE00000000. The full names of the data are: 1) SUBID: SUB7928927, 2). BioProject: PRJNA449941, 3) BioSample: SAMN02414627, SAMN02414631, SAMN10915869, SAMN10915871, SAMN10915877, SAMN15396325, SAMN15396326, SAMN15396328, 4). Accession: GIUE00000000 and 5) Organism: *Pinctada margaritifera*. All codes for RNA-seq analysis are available upon request.

## Abbreviations

DEGs: differentially expressed genes
DNA: Deoxyribonucleic acid
GO: gene ontology
KAAS: KEEG automatic annotation server
KEGG: Kyoto encyclopedia of genes and genomes
MF: Molecular Functions
NCBI: National Center for Biotechnology
PCA: principal component analysis
PE: Paired-end
REVIGO: reduce visualize gene ontology tool
RNA: Ribonucleic acid
RNA-seq: Ribonucleic acid sequencing
SNP: Single nucleotide polymorphism
