# Supplementary material for: Tracing key genes associated with the Pinctada margaritifera albino phenotype from juvenile to cultured pearl harvest stages using multiple whole transcriptome sequencing
Source: BMC Genomics. 2020 Sep 25;21:662. doi: 10.1186/s12864-020-07015-w (PMC7517651; doi:10.1186/s12864-020-07015-w)

**Additional file 4**

**Tracing key genes associated with *Pinctada margaritifera* albino phenotype from juvenile to cultured pearl harvest stages by multiple whole transcriptome sequencing**

P. Auffret, J. Le Luyer, M. Sham Koua, V. Quillien, C-L. Ky

Figure S1.

Figure S1 Summarized REVIGO treemaps plot for gene ontology enrichment analysis between the P. margaritifera albino *versus* black wild-type phenotype

A. Juvenile dataset, UP-regulated genes in Albino phenotype

B. Juvenile dataset, DOWN-regulated genes in Albino phenotype

C. Mantle dataset, UP-regulated genes in Albino phenotype

D. Mantle dataset, DOWN-regulated genes in Albino phenotype

E. Pearl Sac dataset, UP-regulated genes in Albino phenotype

F. Pearl Sac dataset, DOWN-regulated genes in Albino phenotype

G. Common Mantle – Juvenile DOWN-regulated genes in Albino phenotype

H. Common Mantle – Pearl Sac DOWN-regulated genes in Albino phenotype

A.


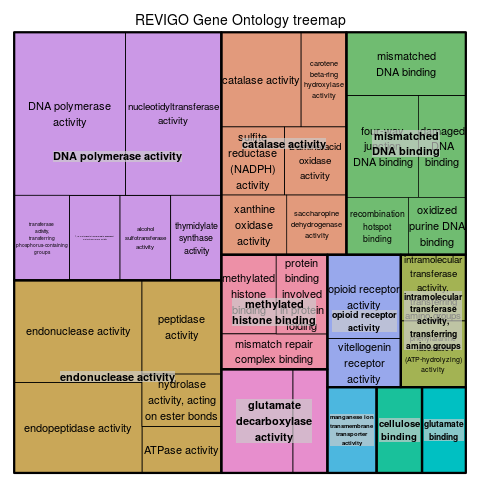


B.


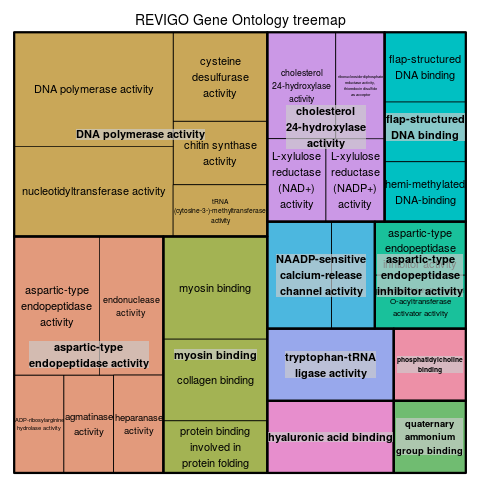


C.


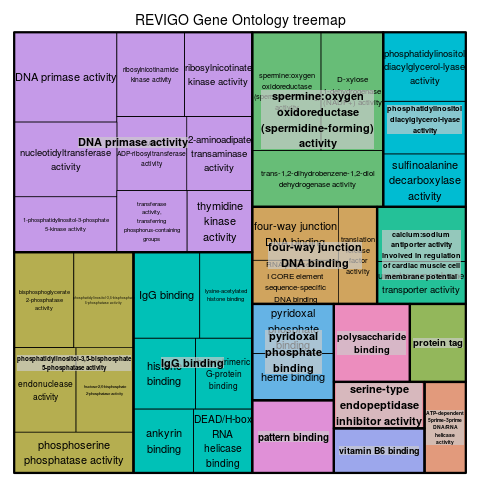


D.


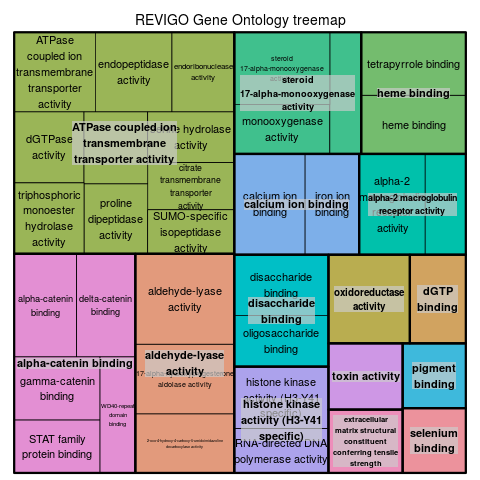


E.


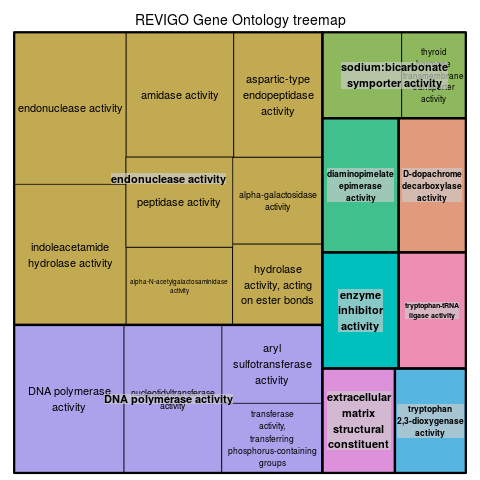


F.


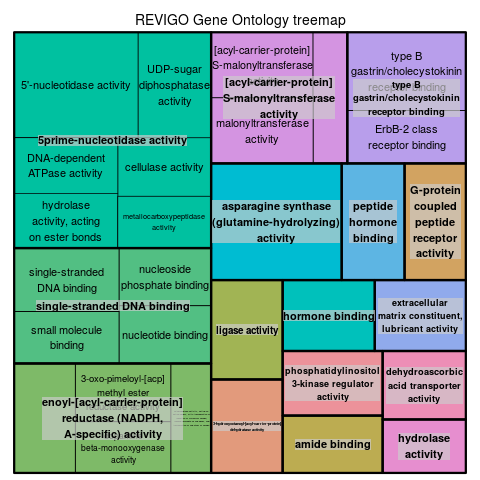


G.


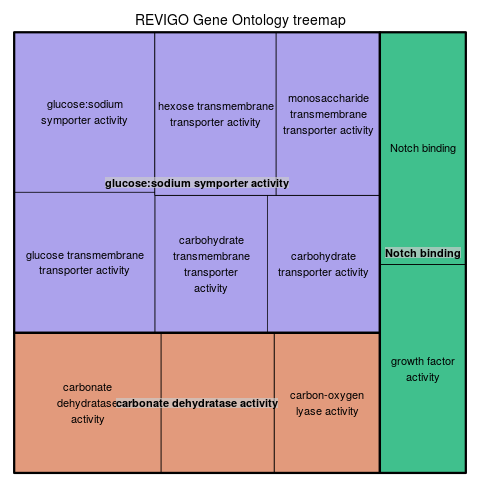


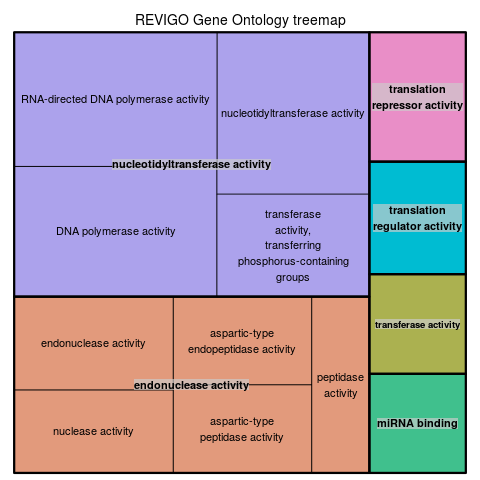
H.

Figure S2.

Figure S2. Signaling pathways (sp) potentially impacted by deregulated genes in albino pearl oyster *P. margaritifera* versus black-wild type

The pathways plots are obtained from KAAS server using DEGs nucleotide sequences. Green boxes show deregulated genes in albino phenotype versus black wild-type in :

A, B, D, E : Mantle dataset

C, F : Juvenile dataset


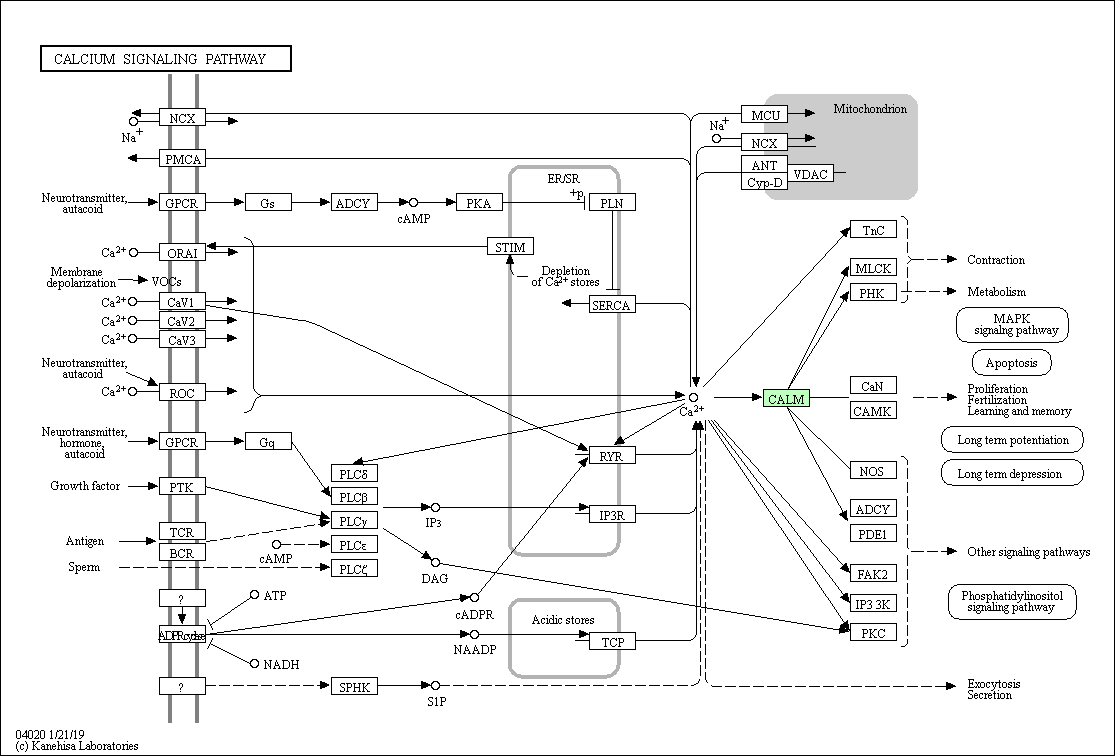


A.


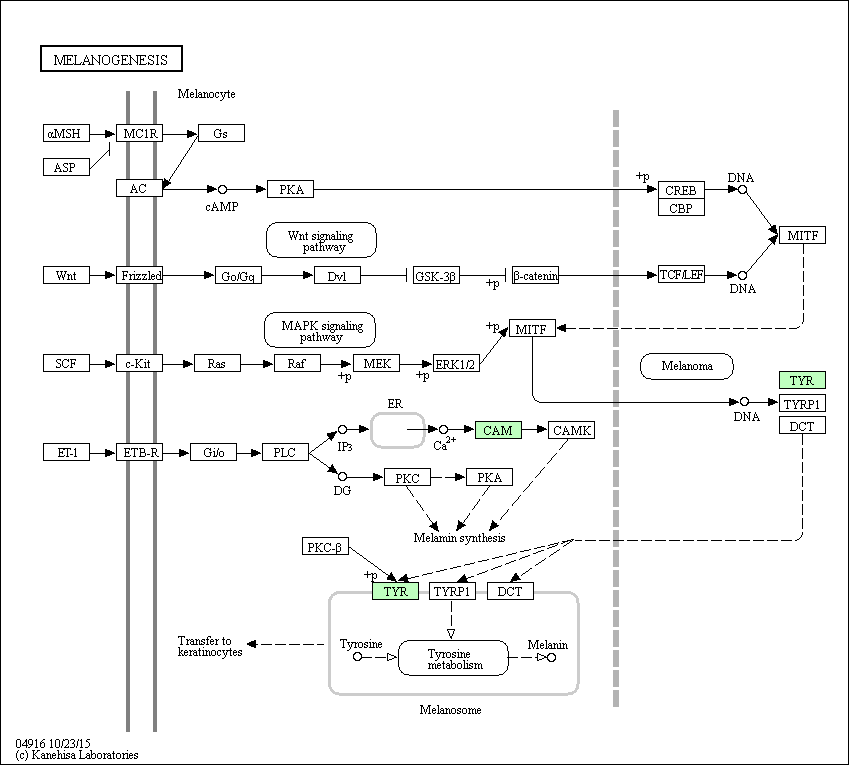


B.


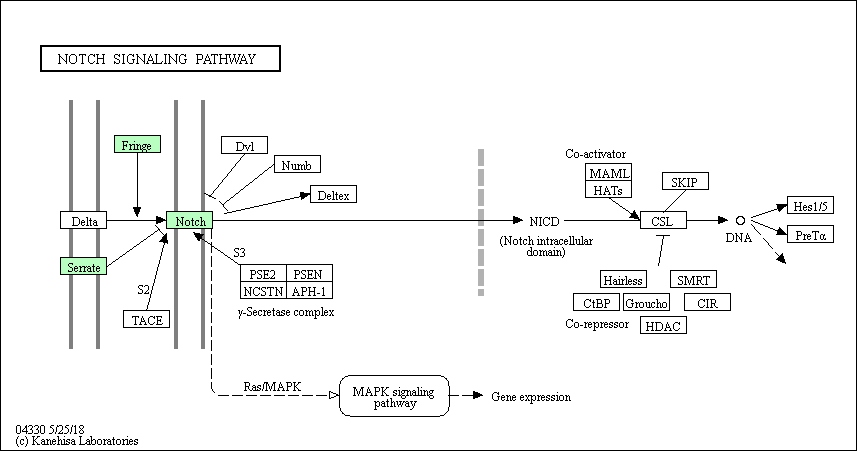


C.


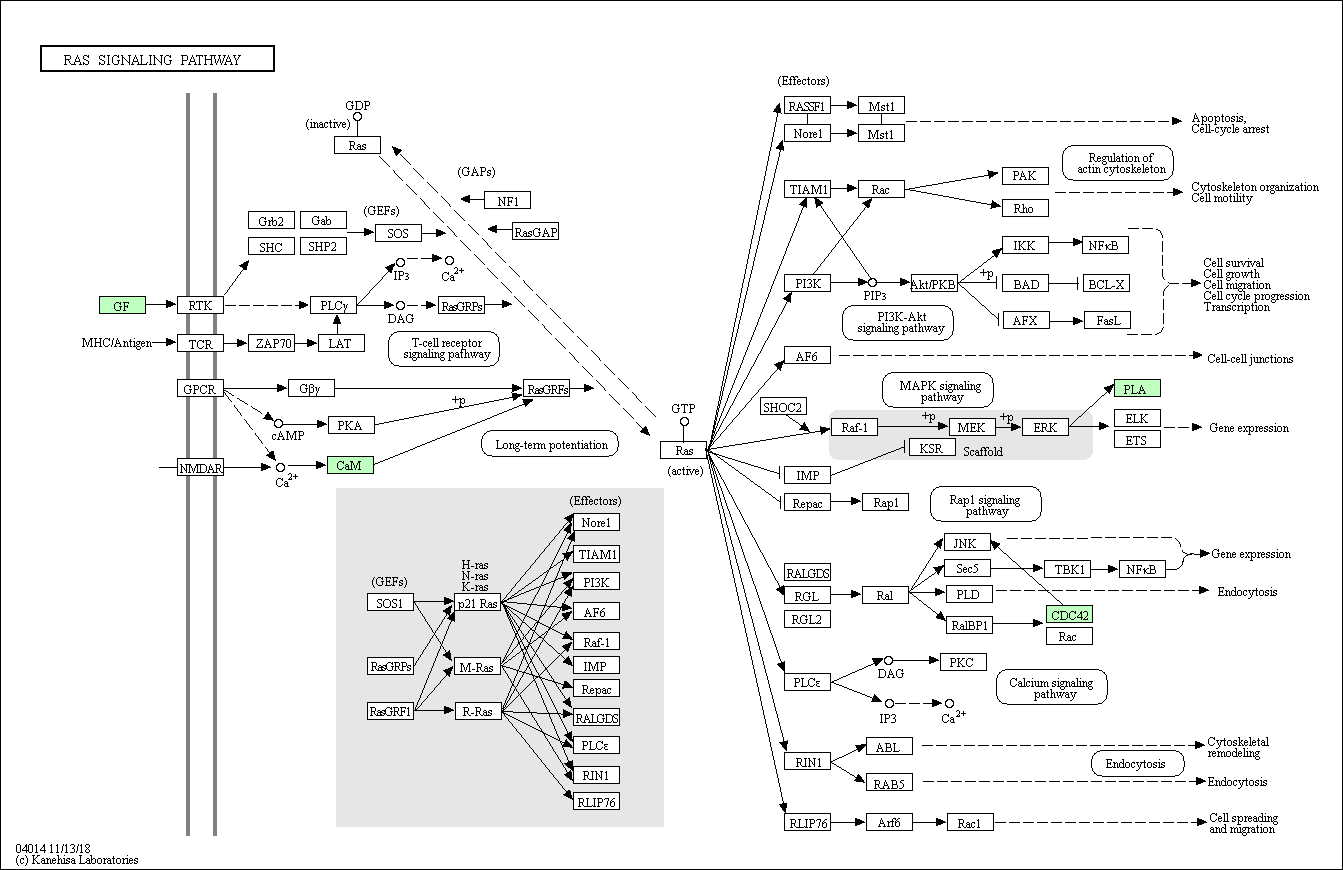


D.


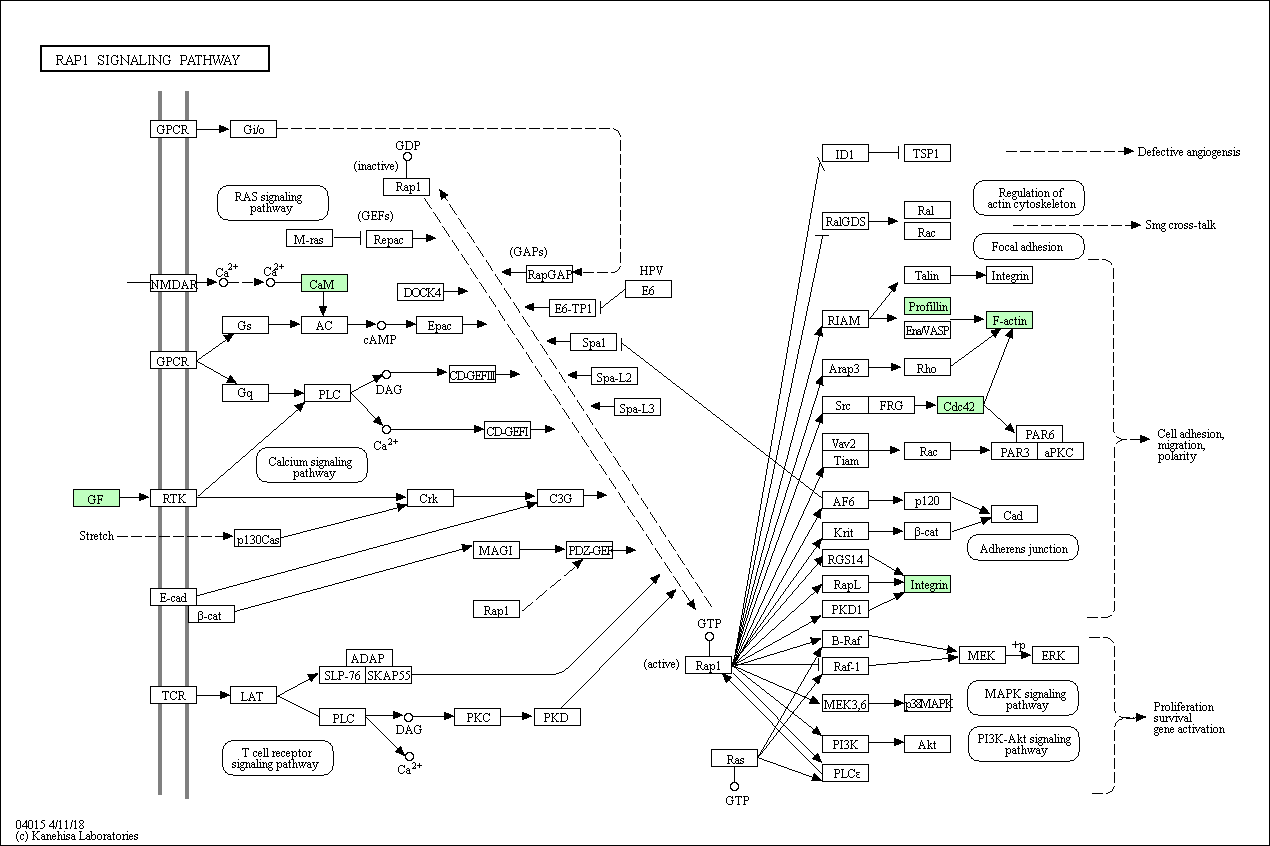


E.


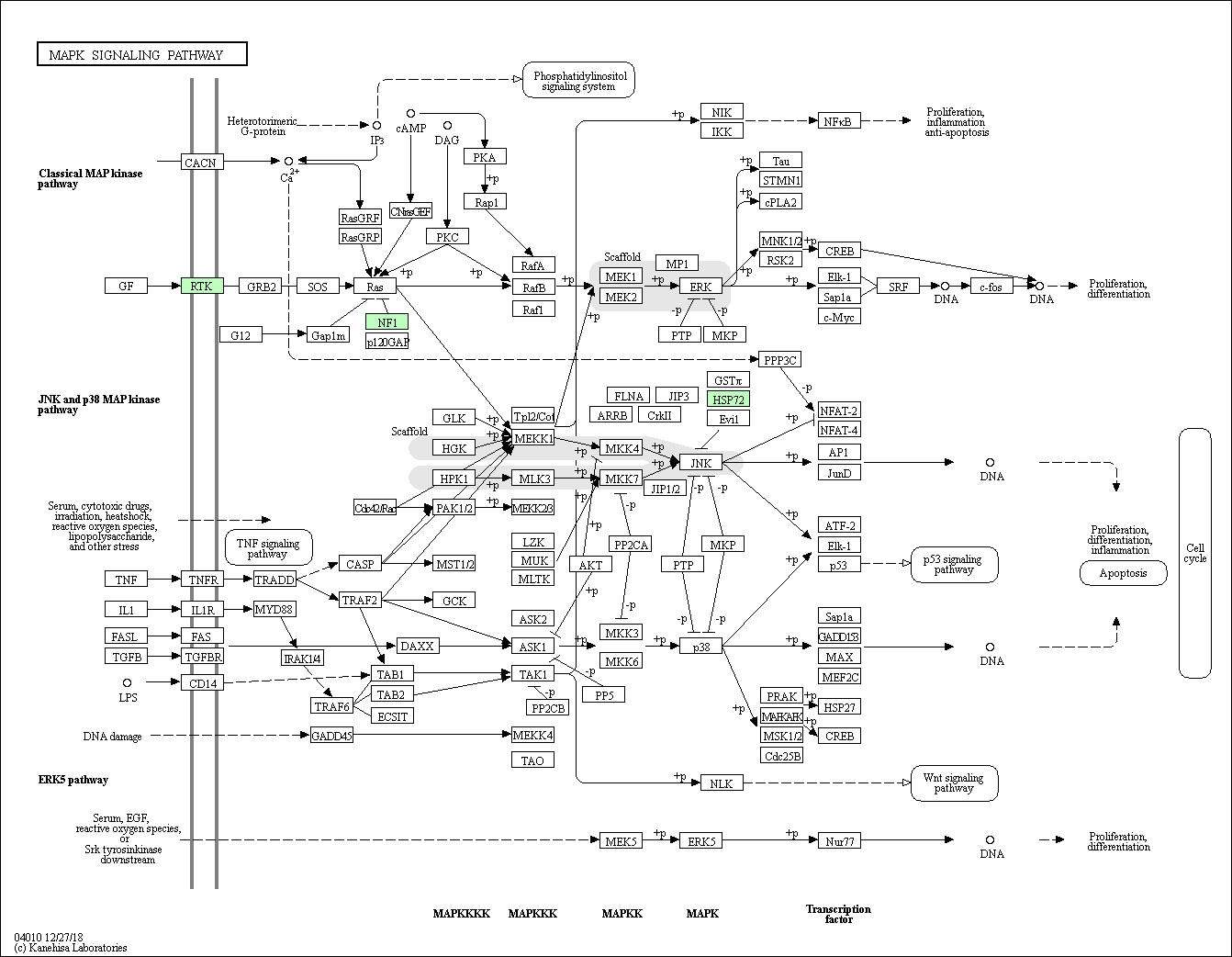


F.

Figure S3.

Figure S3. Composition plots of albino and black wild-type populations of pearl oyster P. margaritifera based on filtered SNPs called with Freebayes in both Juvenile and Mantle datasets


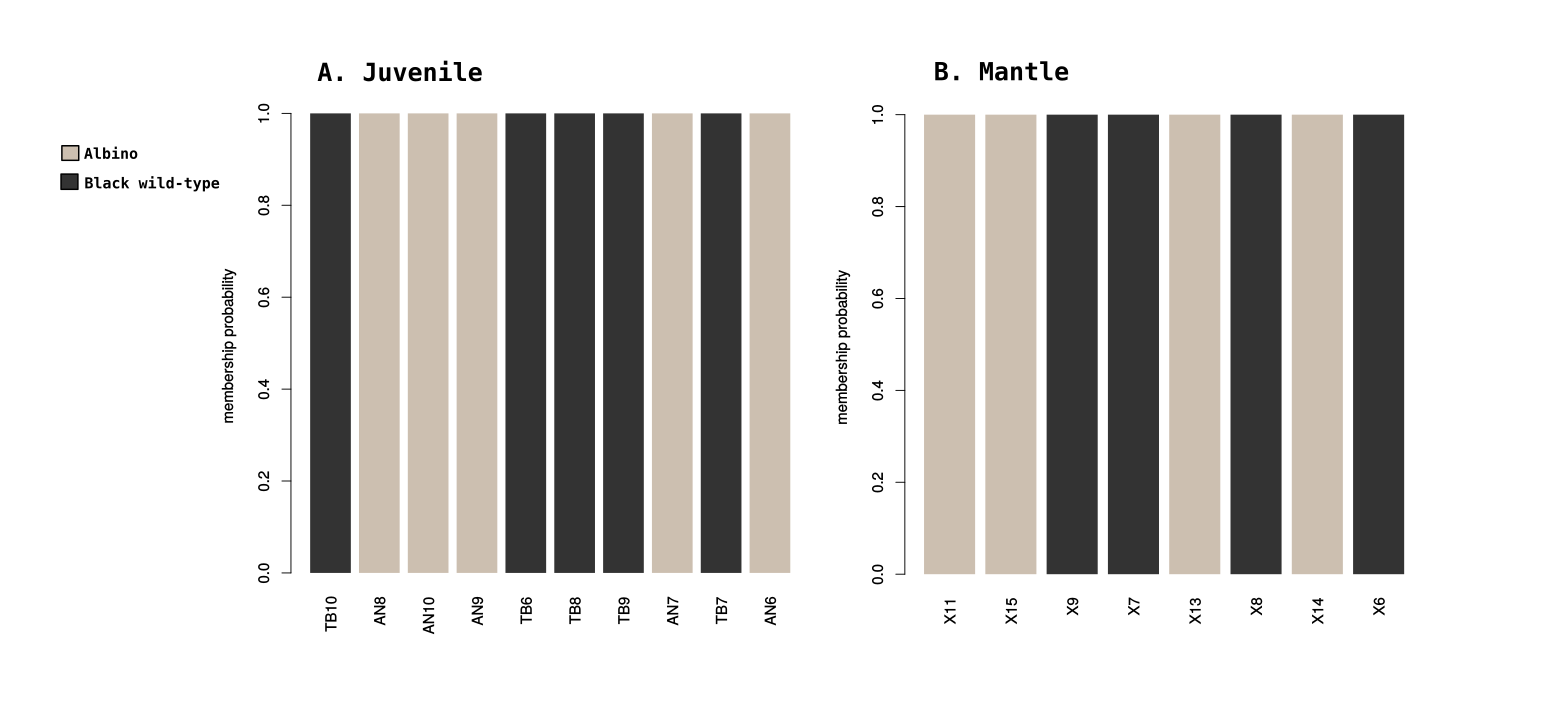

Supplement: Supplementary file 4 — Additional file 4: (.docx) Fig. S1. Summarised REVIGO treemaps plot for gene ontology enrichment analysis between the P. margaritifera albino versus black wild-type phenotypes. Fig. S2. Signalling pathways (sp) potentially impacted by genes deregulated in albino P. margaritifera compared with the black wild-type. Fig. S3. Composition plots of albino and black wild-type populations of pearl oyster P. margaritifera based on filtered SNPs called with Freebayes in both Juvenile and Mantle datasets. [file 12864_2020_7015_MOESM4_ESM.docx]
